# Performance of Bt maize event MON810 in controlling maize stem borers *Chilo partellus* and *Busseola fusca* in Uganda

**DOI:** 10.1016/j.cropro.2022.105945

**Published:** 2022-06

**Authors:** Michael H. Otim, Simon Alibu, Godfrey Asea, Grace Abalo, Julius Pyton Sserumaga, Stella Adumo, Jane Alupo, Stephen Ochen, Tadele Tefera, Anani Y. Bruce, Yoseph Beyene, Barbara Meisel, Regina Tende, Francis Nang'ayo, Yona Baguma, Stephen Mugo, Sylvester O. Oikeh

**Affiliations:** a National Crops Resources Research Institute, Namulonge, P.O. Box 7084, Kampala, Uganda; b National Livestock Resources Research Institute, National Agricultural Research Organization’, P.O. Box 5407, Kampala, Uganda; c International Maize and Wheat Improvement Center (CIMMYT). ICRAF House, United Nations, Avenue, Gigiri P.O. Box 1041, Village Market, 00621, Nairobi, Kenya; d International Centre of Insect Physiology and Ecology (ICIPE), ILRI Campus, Gurd Shola, PO Box 5689, Addis Ababa, Ethiopia; e Bayer Crop Science, 27 Wrench Rd, Isando, Kempton Park, 1600, South Africa; f Kenya Agricultural and Livestock Research Organization, 340-90100, Katumani, Machakos, Kenya; g AATF, P.O. Box 30709-00100, Nairobi, Kenya; h National Agricultural Research Organization, P.O. Box 295, Entebbe, Uganda; i Center for Resilient Agriculture in Africa (CRA-Africa), PO Box 286-00206, Kiserian, Kenya

**Keywords:** *Busseola fusca*, *Chilo partellus*, Bt maize, Exit hole, Stem tunnel, Isolines

## Abstract

Stem borers are major insect pests of maize in Uganda. A study was conducted in 2014–2016 to assess the performance of Bt hybrids expressing Cry1Ab (event MON810) against the two major stem borer species in Uganda – the African stem borer (*Busseola fusca*) and the spotted stem borer (*Chilo partellus*) – under artificial infestation. The study comprised 14 non-commercialized hybrids, including seven pairs of Bt and non-Bt hybrids (isolines), three non-Bt commercial hybrids and a conventional stem borer resistant check. All stem borer damage parameters (leaf damage, number of internodes tunneled and tunnel length) were generally significantly lower in Bt hybrids than in their isolines, the conventionally resistant hybrid, and local commercial hybrids. Mean yields were significantly higher by 29.4–80.5% in the Bt hybrids than in the other three categories of non-Bt hybrids. This study demonstrated that Bt maize expressing Cry1Ab protects against leaf damage and can limit entry of stem borers into the stems of maize plants, resulting in higher yield than in the non-transgenic hybrids. Thus, Bt maize has potential to contribute to the overall management package of stem borers in Uganda.

## Highlights

• Bt maize hybrids significantly reduced stemborer damage.
• Leaf and stem damage were significantly lower in Bt than in non-Bt maize hybrids.
• Bt maize yielded higher than non-Bt maize hybrids by 29.4 to 80.5%.

## 1 Introduction

Stem borers are some of the main insect pests of maize in Uganda. The four major stem borer species are the spotted stem borer, *Chilo partellus* (Swinhoe), the African stem borer *Busseola fusca* (Fuller), the sugarcane borer *Eldana saccharina* (Walker), and the pink stem borer *Sesamia calamistis* (Hampson). *Busseola fusca* and *C. partellus* are the two most widely distributed and dominant species in Uganda (Matama-Kauma et al., 2007; Molo et al., 2014). The larvae of stem borers feed on the plant whorl and tunnel stems leading to the death of growing shoots (Ampofo et, 1986). In Kenya, stem borers were reported to cause losses ranging from 10 to 100% (De Groote et al., 2002; Ong'amo et al., 2006; Seshu Reddy, 1990). Furthermore, in Kenya, total losses to stem borers were valued at USD 25 and USD 59.8 million in 1999 and 2000, respectively (De Groote et al., 2002). In Uganda, yield losses due to stem borers were estimated at 23.5% in 2015 (Wamatsembe et al., 2017). Stem borer damage on maize ears predisposes the grain to pre-harvest infestations by storage insect pests and mycotoxins (Njeru et al., 2020; Opoku et al., 2019)*.* Mycotoxins, including aflatoxins, pose a serious health threat to humans and livestock and are associated with liver cancer, stunted growth in children, and immune disorders (Wu, 2014); these health risks make aflatoxin-contaminated maize grain unsuitable for food and feed.

Cropping system, chemical, cultural, biological, and host plant resistance are used to control stem borers in maize (Khan et al., 1997; Ndemah et al., 2007; Schulthess et al., 1997). The push-pull technology is one of the cropping systems that are effective in controlling stem borers. However, it is poorly adopted by smallholder farmers because it is knowledge- and labor-intensive. In addition, limited availability and high cost of Desmodium seed, a key component of the technology, limits its uptake (Mukebezi, 2008). Spraying with insecticides only protects against early infestations, but not against stem borers feeding inside the ears and stems (Jotwani, 1983). In addition, insecticide use is not cost-effective in smallholder systems and may expose farmers to health and environmental risks. Biological control agents of stem borers have been introduced and released into farmers’ fields but they take long to establish and only provide partial control (Bonhof et al., 1997; Bruce et al., 2009; Schulthess et al., 1997; Zhou et al., 2001). Many farmers have therefore resorted to using insecticides or not controlling stem borers at all. Although host plant resistance is safe and averts the need for farmers to purchase and apply insecticides, not a single stem borer resistant maize variety has been released and commercialized in Uganda, despite the existence of conventional stemborer resistant maize germplasm (CIMMYT, 1993; KEPHIS, 2022). In Kenya, 13 stem borer tolerant/resistant varieties have so far been released (KEPHIS, 2022).

Transgenic plants expressing toxins from *Bacillus thuringiensis* (Bt) with resistance to different groups of insects have been developed by genetically engineering (Koziel et al., 1993; Vaeck et al., 1988). Transgenic Bt maize can help to control several species of Lepidopteran stem borers, e.g. *Ostrinia nubilalis* (Hübner) (Magg et al., 2001), *S. calamistis* (Van Den Berg and Van Wyk, 2007), *Sesamia nonagrioides* (Lefèbvre) (Farinós et al., 2011), *B. fusca* and *C. partellus* (Tefera et al., 2016; Tende et al., 2010). The Bt toxin *Cry1Ab* included in event MON810 was deregulated and commercialized in the United States in 1996. It has also been approved for importation and cultivation in many countries in Latin America, Asia, and Europe (ISAAA, 2017). Farmers in South Africa started growing MON810 maize hybrids in 1998 (Kruger et al., 2012), and later Bt11 (also Cry1Ab) and MON89034 (Cry1A.105 + Cry2Ab2) were approved for control of stem borers in 2003 and 2010, respectively (De Buck et al., 2016). In Egypt, MON810 was approved for cultivation in 2008 (Sawahel, 2008). To date, Bt maize has not been approved for commercial use in Uganda.

No studies have been conducted on the effectiveness of Bt maize in controlling stem borers in Uganda, yet the expression and efficacy of Cry1Ab protein can vary with environmental conditions and farming systems/practice (Nguyen and Jehle, 2007; Székács et al., 2010; Trtikova et al., 2015). There are also known Cry1Ab resistant strains of *B. fusca* in South Africa (Campagne et al., 2013). The present study was conducted for three seasons under the supervision of the Uganda National Biosafety Committee (NBC) to generate empirical information to inform decision making on application for approval of Bt maize cultivation in Uganda. The performance of the Bt maize event MON810 in controlling two stem borer species (*C. partellus* and *B. fusca*) under artificial infestation was evaluated in confined field trials (CFTs) using different maize genotypes.

## 2 Materials and methods

### 2.1 Study location

The experiments were conducted in confined field trials (CFTs) following the national guidelines (UNCST, 2006). One CFT site was located at the National Crops Resources Research Institute (NaCRRI), Namulonge, Wakiso district (0.525931, 32.622453), and the other in Mubuku irrigation and settlement scheme in Kasese district (0.20845, 30.12483). National Crops Resources Research Institute, a constituent Institute of the Ugandan National Agricultural Research Organization with a mandate for maize research, has a well-developed CFT village with biosafety facilities required for the study. These trials were part of research under the Water Efficient Maize for Africa (WEMA) partnership coordinated by African Agriculture Technology Foundation (AATF). The other partners included Monsanto, International Maize and Wheat Improvement Centre (CIMMYT) and National Agricultural Research Systems in Kenya, Tanzania, Mozambique and South Africa.

NaCRRI oversees maize research in Uganda and was, therefore, chosen because of the existing technical and physical resources, including a well-developed CFT village required for the study. The Institute is 27 km north of Kampala, on the Kampala-Gayaza-Zirobwe road. The rainfall pattern at NaCRRI is bi-modal with peaks in April and October. Average rainfall ranges from 900 mm to 1,200 mm per annum.

Mubuku irrigation scheme in Kasese was chosen because it hosts one of the largest and oldest CFT sites in the country and has a well-developed irrigation system for off-season planting. Mubuku lies at an altitude of about 1,007 m asl, with a mean annual temperature and rainfall of 27.8 °C and 750 mm, respectively.

### 2.2 Germplasm

Genotypes in the field trials included seven Bt hybrids (maize hybrids with event MON810 expressing insecticidal Cry1Ab protein) and seven of their corresponding non-transgenic near-isogenic hybrids (isolines). The isolines are non-GM hybrids from inbred lines with genetic background identical to those of their corresponding GM lines. Three commercial non-transgenic maize varieties sourced from seed companies (East African Seeds, Farm Input Care Centre and Nalweyo Seed Company) in Kampala and a conventionally bred *B. fusca* and *C. partellus* resistant hybrid from CIMMYT (Munyiri et al., 2013) were also used (Table 1). The Bt hybrids and isolines were sourced from Monsanto Company (now Bayer).

**Table 1:** List of Bt maize hybrids, non-Bt isolines commercial checks and resistant check used in the experiment.

Entry code (Bt hybrids)	Entry code (non-Bt hybrids)	Commercial checks	Resistant check
Hybrid 1 – MON810	Hybrid 2 – Isoline	Longe 10H	CIMMYT conv. resistant hybrid (CKIR06009)
Hybrid 3 – MON810	Hybrid 4 – Isoline	DK 8031	
Hybrid 5 – MON810	Hybrid 6 – Isoline	Longe 6H	
Hybrid 7 – MON810	Hybrid 8 – Isoline		
Hybrid 9 – MON810	Hybrid 10 – Isoline		
Hybrid 11 – MON810	Hybrid 12 – Isoline		
Hybrid 13 – MON810	Hybrid 14 – Isoline		

### 2.3 Experimental design

The experiments were conducted from 2014 to 2016. *Chilo partellus* trials were planted at NaCRRI on January 6, 2014 and December 19, 2014 and harvested on May 25, 2014 and May 11, 2015, respectively. The *B. fusca* trial was planted in Mubuku, Kasese on August 28, 2015 and harvested on January 15, 2016. Choice of the sites for evaluating the two species depended on an earlier report that *B. fusca* and *C. partellus* were more abundant in the western mid altitude farmlands and Semeliki flats (Kasese) and Lake Victoria Crescent (Wakiso), respectively (Molo et al., 2014), and the presence of well-developed CFTs. In order to provide temporal isolation, the planting times were chosen to coincide with a period when most other maize crops in the vicinity were almost mature. This, in addition to implementing spatial isolation, was meant to avoid pollen exchange between Bt and non-Bt maize. The trials were planted in an alpha lattice experimental design, nine entries by two blocks, with four replications. There were two-row plots per entry. Two seeds were planted per hill in a row of 5 m length and thinned to one seedling per hill at two weeks after emergence. This made a total of 21 plants per row. The inter- and intra-row plant spacing was 75 cm and 25 cm, respectively, giving a population of 53,333 plants ha‾^1^. Standard rates of fertilizers were applied (125 kg N and 125 kg P_2_O_5_ ha‾^1^). Top dressing was done using urea in two splits. Supplemental irrigation was applied when needed. The fields were kept weed-free by hand weeding. The non-infested plants in each row were protected with Bulldock® 25 EC (Beta-cyfluthrin), but data were not collected from the sprayed plants.

### 2.4 Stem borer rearing and infestation

The rearing of *C. partellus* and *B. fusca* followed methods of Tefera et al. (2016). The stem borers were reared in an insectary at NaCRRI from field-collected populations of the stem borers. To ensure that neonates were alive at the time of infestation, both eggs and larvae were handled carefully to ensure maximal survival. The eggs were collected, surface sterilized using formaldehyde, and dried on filter paper as described by Tefera et al. (2016). The collected eggs were then kept under room temperature (26 ± 2 °C) for 4–5 days to develop into the blackhead stage. The eggs were then monitored for 1–2 days, within which period they hatched and were used for infestation. Where the number of neonates was low, further development of the emerged neonates was delayed by subjecting them to a temperature of 4 °C until enough neonates were recovered for infestation.

Five plants, from the second to sixth plant in each row (10 plants per plot), were infested with 10 neonates of the target stemborer species (*C. partellus* at NaCRRI and *B. fusca* in Kasese), starting at about three weeks after the emergence of maize plants and repeated at weekly intervals. A total of three infestations was used for *C. partellus*. For the *B*. *fusca* experiment, the infestation was done four times because the required number of neonates could not be obtained; infestations used for *B. fusca* were 10, 3, 6 and 10 neonates per plant for the first, second, third and fourth infestations, respectively.

A camel-hair brush was used to transfer the neonates into the young maize whorls. Only active neonates were used to infest the maize plants between 8:30 a.m. and 12:00 p.m., and 4:00 p.m.–5:30 p.m. to avoid exposing the neonates to harsh sunny conditions, which could lead to desiccation. The neonates were placed directly into the cooler and concealed maize whorls.

### 2.5 Data collection

Data on stem borer damage were collected on the leaves, internodes and stems (tunnel length) of maize. Leaf damage by stem borers was assessed by scoring each infested plant on a scale of 1–9 (where 1 = no visible damage and 9 = completely damaged) (Tefera et al., 2011). Scores of leaf damage were taken three times fortnightly beginning at two weeks after infestation. At harvest, the 10 infested plants were stripped off the leaves and assessed for stem damage. The number of stem borer exit holes per plant and the number of tunneled internodes were counted and recorded. The stalks were then split open to record the tunnel length, the number of larvae and number of pupae.

To determine grain yield, only the infested plants (10 plants per plot) were harvested. The ears from the harvested plants were weighed separately and their respective moisture content was determined from a sample of grain. The yield was then converted to grain yield per hectare, assuming 80% shelling percentage and adjusted for moisture content at 13.5% using the formula below:

$$Dry\;grain\;weight\;\left(kg\right)=Field\;weight*\frac{\left(100-\%\;\text{Field MC}\right)}{86.5}$$

Where MC = Moisture content.

### 2.6 Statistical analysis

The means of the different data parameters were calculated for each experimental unit in Microsoft Excel. Before analysis, all data were checked for the assumption of normality and homogeneity of variance using GenStat (International, n.d.). The number of exit holes and internodes tunneled, as well as tunnel lengths, were not normally distributed and were, therefore, transformed using square root transformation since they all had several zero counts and measurements. All data were analyzed using ANOVA, with contrasts for pairwise comparison between Bt and their non-Bt isolines. Similarly, we used ANOVA with contrasts for comparing the Bt hybrids, non-Bt Isolines, resistant check, and commercial checks. The means were compared using multiple comparison tests using Fisher's LSD method. All statistical analyses were done using GenStat V12.1.3338 (International, n.d.). Untransformed data are presented in the results.

## 3 Results

### 3.1 Effect of Bt maize and non-Bt maize on leaf, stem damage and grain yield

There were significant differences between Bt- and isolines in leaf damage in both seasons for *C. partellus* (Table 2). Mean leaf damage scores were significantly lower in all Bt hybrids when compared with their isolines. Mean leaf damage ranged from 1 to 1.4 in Bt hybrids and from 4.6 (entry 10) to 6.1 (entry 14) in isolines. Similar trends were observed for *B. fusca* in leaf damage for both Bt- and isolines (Table 2). *Busseola fusca* leaf damaged score ranged from 1.7 to 1.9 in Bt hybrids, and from 3 to 3.5 in isolines. The number of exit holes followed as similar pattern as leaf damage, being significantly lower in all Bt hybrids than in the isolines in the two *C. partellus* plantings, and in six of the seven Bt/isoline combinations (Table 3). Hybrids 8 and 12 recorded the highest exit holes in both seasons of infestation with *C. partellus*. Similarly, significant differences were observed in number of internodes tunneled (Table 4) and tunnel length (Table 5). Most of the Bt hybrids were highly resistant to tunneling caused by *C. partellus* in both seasons. There were, however, longer tunnels recorded in *B. fusca* infested plants (hybrids 4, 8, 10, 14) than in *C. partellus* infested plants (Table 5). For grain yield, there were significant differences between Bt-hybrids and isolines under both *C. partellus* and *B. fusca* infestation (Table 6). All Bt hybrids had greater yields than isolines in the *C. partellus* infested experiments. Among the *C. partellus* infested hybrids, hybrids 3, 5 and 7 had the highest yield (8.5–8.6 kg/ha) in the first season and hybrids 1, 3, 5, 7 and 13 had the highest yields (10.5–12 kg/ha) in the second season. In the *B. fusca* experiment, significant differences occured between the Bt and isoline pairs in grain yield only between Bt hybrid 13 (9.1 kg/ha) and isoline 14 (7.3 kg/ha). All the other comparisons between Bt and isolines in the *B. fusca* infested plants were not significantly different.

**Table 2:** Mean leaf damage (±SEM) of Bt maize and non-Bt isolines following artificial infestation with *Chilopartellus* at Namulonge, Wakiso (Season 1 and 2), and with *Busseolafusca* at Mubuku, Kasese (Season 3), Uganda from 2014 to 2016 crop season.

Entry code	Mean leaf damage score			
	C. partellus			B. fusca
	Season 1	Season 2	Mean	Season 1
Hybrid 1 – MON810	1.0 ± 0.00a	1.0 ± 0.00a	1.0 ± 0.00a	1.8 ± 0.08a
Hybrid 2 – Isoline	4.9 ± 0.60b	4.9 ± 0.39b	4.9 ± 0.33b	3.0 ± 0.24b
Hybrid 3 – MON810	1.0 ± 0.01a	1.0 ± 0.02a	1.0 ± 0.01a	1.7 ± 0.06a
Hybrid 4 – Isoline	4.8 ± 0.42b	4.7 ± 0.19b	4.7 ± 0.21b	3.1 ± 0.14b
Hybrid 5 – MON810	1.2 ± 0.13a	1.4 ± 0.26a	1.3 ± 0.14a	1.9 ± 0.10a
Hybrid 6 – Isoline	5.2 ± 0.40b	5.4 ± 0.39b	5.3 ± 0.26b	3.4 ± 0.17b
Hybrid 7 – MON810	1.0 ± 0.01a	1.0 ± 0.00a	1.0 ± 000a	1.9 ± 0.09a
Hybrid 8 – Isoline	4.6 ± 0.28b	5.2 ± 0.22b	4.9 ± 0.19b	3.3 ± 0.33b
Hybrid 9 – MON810	1.1 ± 0.13a	1.2 ± 0.13a	1.2 ± 0.08a	1.9 ± 0.10a
Hybrid 10 – Isoline	4.6 ± 0.46b	5.5 ± 0.17b	5.1 ± 0.28b	3.2 ± 0.24b
Hybrid 11 – MON810	1.0 ± 0.02a	1.0 ± 0.03a	1.0 ± 0.02a	1.9 ± 0.08a
Hybrid 12 – Isoline	4.9 ± 0.21b	5.5 ± 0.29b	5.2 ± 0.19b	3.4 ± 0.34b
Hybrid 13 – MON810	1.3 ± 0.15a	1.4 ± 0.35a	1.3 ± 0.18a	1.8 ± 0.06a
Hybrid 14 – Isoline	6.1 ± 0.40b	5.1 ± 0.18b	5.6 ± 0.27b	3.5 ± 0.17b
F_13,39_	53.88	13.58	121.93	83.35
P-Value	<0.001	<0.001	<0.001	<0.001
				

Entries with odd numbers have Bt genes and those with even numbers do not have the Bt gene (isolines). Each pair of means within a column followed by different letters are significantly different.

**Table 3:** The number of exit holes (±SEM) of Bt hybrids and their non-Bt isolines following artificial infestation with *Chilopartellus* at Namulonge, Wakiso, and with *Busseolafusca* at Mubuku, Kasese, Uganda from 2014 to 2016 crop season.

**Entry code**	Mean number of exit holes			
	C. partellus			B. fusca
	**Season 1**	**Season 2**	**Mean**	**Season 1**
Hybrid 1 – MON810	0.4 ± 0.24a	0.0 ± 0.00a	0.2 ± 0.13a	0.0 ± 0.00a
Hybrid 2 – Isoline	5.7 ± 1.28b	7.2 ± 0.87b	6.5 ± 0.77b	1.2 ± 0.49b
Hybrid 3 – MON810	0.0 ± 0.03a	0.0 ± 0.03a	0.0 ± 0.02a	0.2 ± 0.09a
Hybrid 4 – Isoline	2.3 ± 0.77a	4.0 ± 0.47b	3.1 ± 0.53b	1.7 ± 0.47b
Hybrid 5 – MON810	0.2 ± 0.09a	0.1 ± 0.11a	0.1 ± 0.07a	0.2 ± 0.13a
Hybrid 6 – Isoline	6.3 ± 2.12b	4.3 ± 0.900b	5.3 ± 1.13b	0.7 ± 0.32a
Hybrid 7 – MON810	0.5 ± 0.29a	0.0 ± 0.00a	0.3 ± 0.17a	0.1 ± 0.08a
Hybrid 8 – Isoline	8.0 ± 2.28b	9.5 ± 1.90b	8.8 ± 1.40b	3.3 ± 0.98b
Hybrid 9 – MON810	0.1 ± 0.11a	0.0 ± 0.00a	0.1 ± 0.06a	0.1 ± 0.01a
Hybrid 10 – Isoline	4.9 ± 1.21b	4.1 ± 0.45b	4.5 ± 0.61b	1.4 ± 0.32b
Hybrid 11 – MON810	0.1 ± 0.06a	0.1 ± 0.01a	0.1 ± 0.04a	0.0 ± 0.00a
Hybrid 12 – Isoline	8.3 ± 1.31b	8.6 ± 0.26b	8.4 ± 0.62b	1.6 ± 0.50b
Hybrid 13 – MON810	0.0 ± 0.00a	0.8 ± 0.75a	0.4 ± 0.38a	0.0 ± 0.03a
Hybrid 14 – Isoline	2.6 ± 0.74a	7.0 ± 1.73b	4.8 ± 1.19b	2.0 ± 0.53b
F_13,39_	15.94	37.45	40.4	8.68
P-Value	<0.001	<0.001	<0.001	<0.001

Entries with odd numbers have the Bt genes and those with even numbers do not have the Bt genes (isolines). Each pair of means within a column followed by different letters are significantly different at P < 0.05 using t-tests.

**Table 4:** Mean number of internodes tunneled (±SEM) of Bt hybrids and their isolines following artificial infestation with *Chilopartellus* at Namulonge, Wakiso, and with *Busseolafusca* at Mubuku, Kasese, Uganda from 2014 to 2016 crop season.

Entry code	Mean number of internodes tunneled			
	C. partellus			B. fusca
	Season 1	Season 2	Mean	Season 3
Hybrid 1 – MON810	0.4 ± 0.24a	0.0 ± 1.380	0.2 ± 0.13a	0.0 ± 0.00a
Hybrid 2 – Isoline	2.9 ± 0.79b	2.3 ± 0.19b	2.6 ± 0.39b	0.3 ± 0.21a
Hybrid 3 – MON810	0.0 ± 0.03a	0.0 ± 0.03a	0.0 0.02a	0.1 ± 0.06a
Hybrid 4 – Isoline	1.1 ± 0.40a	1.6 ± 0.12b	1.4 ± 0.22b	1.0 ± 0.31b
Hybrid 5 – MON810	0.1 ± 0.06a	0.1 ± 0.04a	0.1 ± 0.03a	0.1 ± 0.11a
Hybrid 6 – Isoline	3.1 ± 0.76b	1.8 ± 0.23b	2.5 ± 0.45b	0.5 ± 0.22a
Hybrid 7 – MON810	0.43 ± 0.16a	0.0 ± 0.00a	0.2 ± 0.11a	0.0 ± 0.03a
Hybrid 8 – Isoline	3.0 ± 0.29b	3.0 ± 0.61b	3.0 ± 0.31b	1.2 ± 0.73b
Hybrid 9 – MON810	0.1 ± 0.11a	0.0 ± 0.00a	0.1 ± 0.06a	0.0 ± 0.00a
Hybrid 10 – Isoline	3.2 ± 1.05b	1.6 ± 0.11b	2.4 ± 0.57b	0.5 ± 0.34a
Hybrid 11 – MON810	0.1 ± 0.06a	0.1 ± 0.05a	0.1 ± 0.04a	0.0 ± 0.00a
Hybrid 12 – Isoline	3.4 ± 0.31b	2.9 ± 0.27b	3.2 ± 0.21b	0.7 ± 0.31a
Hybrid 13 – MON810	0.0 ± 0.00a	0.2 ± 0.18a	0.1 ± 0.09a	0.0 ± 0.03a
Hybrid 14 – Isoline	2.2 ± 0.57b	2.8 ± 0.65b	2.5 ± 0.41b	0.7 ± 0.17a
F_13,39_	13.21	42.14	35.99	2.59
P-Value	<0.001	<0.001	<0.001	<0.001

*Entries with odd numbers have the Bt gene and those with even numbers do not have the Bt gene (isolines). Each pair of means within a column followed by different letters are significantly different*.

**Table 5:** Mean tunnel length (±SEM) of Bt hybrids and their non-Bt isolines following artificial infestation with *Chilopartellus* at Namulonge, Wakiso and with *Busseolafusca* at Mubuku, Kasese, Uganda from 2014 to 2016 Crop season.

**Entry code**	Mean length of tunnels (cm)			
	C. partellus			B. fusca
	**Season 1**	**Season 2**	**Mean**	**Season 3**
Hybrid 1 – MON810	0.9 ± 0.72a	0.0 ± 0.00a	0.4 ± 0.37a	0.0 ± 0.00a
Hybrid 2 – Isoline	3.5 ± 0.57b	4.4 ± 0.68b	4.0 ± 0.45b	7.0 ± 2.35b
Hybrid 3 – MON810	0.1 ± 0.05a	0.0 ± 0.03a	0.0 ± 0.03a	5.3 ± 2.96a
Hybrid 4 – Isoline	1.8 ± 0.67b	3.0 ± 0.31b	2.4 ± 0.41b	11.6 ± 2.76b
Hybrid 5 – MON810	0.2 ± 0.15a	0.1 ± 0.07a	0.1 ± 0.08a	4.0 ± 2.43a
Hybrid 6 – Isoline	4.7 ± 1.61b	2.9 ± 0.52b	3.8 ± 0.85b	9.3 ± 0.27b
Hybrid 7 – MON810	1.0 ± 0.38a	0.0 ± 0.00a	0.5 ± 0.26a	2.7 ± 2.72a
Hybrid 8 – Isoline	3.7 ± 0.63b	5.1 ± 1.29b	4.4 ± 0.72b	10.5 ± 2.75b
Hybrid 9 – MON810	0.1 ± 0.15a	0.0 ± 0.00a	0.1 ± 0.07a	2.3 ± 2.31a
Hybrid 10 – Isoline	3.4 ± 0.49b	3.5 ± 0.34b	3.5 ± 0.28b	10.7 ± 2.26b
Hybrid 11 – MON810	1.1 ± 0.706a	0.2 ± 0.15a	0.6 ± 0.38a	0.0 ± 0.00a
Hybrid 12 – Isoline	3.7 ± 0.31b	4.7 ± 1.40b	4.2 ± 0.69b	9.8 ± 0.62b
Hybrid 13 – MON810	0.0 ± 0.00a	0.6 ± 0.57a	0.3 ± 0.28a	0.5 ± 0.50a
Hybrid 14 – Isoline	2.3 ± 0.35b	3.7 ± 0.78b	3.0 ± 0.47b	12.5 ± 1.93b
F_13,39_	10.39	26.71	27.8	6.27
P-Value	<0.001	<0.001	<0.001	0.011

Entries with odd numbers have the Bt gene and those with even numbers do not have the Bt gene (isolines). Each pair of means within a column followed by different letters are significantly different.

**Table 6:** Mean grain yield (±SEM) of Bt hybrids and their isolines following artificial infestation with *Chilopartellus* at Namulonge, Wakiso, and with *Bsseolafusca* at Mubuku, Kasese, Uganda from 2014 to 2016 crop season.

**Entry code**	Mean grain yield (t ha^−1^) under different stem borer species			
	C. partellus			B. fusca
	**Season 1**	**Season 2**	**Mean**	**Season 1**
Hybrid 1 – MON810	7.5 ± 0.73b	10.5 ± 0.54b	9.0 ± 0.71b	8.6 ± 0.69a
Hybrid 2 – Isoline	4.7 ± 0.92a	6.6 ± 0.79a	5.7 ± 0.66a	8.0 ± 0.35a
Hybrid 3 – MON810	8.5 ± 0.60b	12.0 ± 0.12b	10.1 ± 0.78b	8.6 ± 0.62a
Hybrid 4 – Isoline	5.5 ± 1.03a	9.3 ± 0.41a	7.4 ± 0.90a	8.9 ± 0.47a
Hybrid 5 – MON810	8.6 ± 0.74b	11.0 ± 0.57b	9.8 ± 0.62b	9.0 ± 0.71a
Hybrid 6 – Isoline	3.7 ± 0.69a	6.4 ± 1.41a	5.0 ± 0.88a	8.1 ± 0.37a
Hybrid 7 – MON810	8.5 ± 0.71b	11.4 ± 0.35b	10.0 ± 0.66b	9.3 ± 0.62a
Hybrid 8 – Isoline	5.4 ± 0.79a	6.1 ± 0.76a	5.7 ± 0.52a	9.7 ± 0.29a
Hybrid 9 – MON810	6.8 ± 0.90b	9.4 ± 0.19b	8.1 ± 0.65b	8.4 ± 0.29a
Hybrid 10 – Isoline	4.6 ± 0.98a	4.0 ± 1.22a	4.3 ± 0.73a	8.2 ± 0.41a
Hybrid 11 – MON810	7.5 ± 0.45b	8.5 ± 0.17b	7.9 ± 0.26b	9.2 ± 0.68a
Hybrid 12 – Isoline	4.6 ± 0.41a	4.4 ± 0.73a	4.5 ± 0.39a	8.7 ± 0.73a
Hybrid 13 – MON810	7.6 ± 1.00b	11.8 ± 0.79b	9.7 ± 0.98b	9.1 ± 0.23b
Hybrid 14 – Isoline	2.5 ± 0.71a	6.2 ± 0.93a	4.4 ± 0.88a	7.3 ± 0.53a
F_13,39_	9.22	13.55	10.0	2.53
P-Value	<0.001	<0.001	<0.001	0.013

Entries with odd numbers have the Bt gene and those with even numbers do not have the Bt gene (isolines). Each pair of means within a column followed by different letters are significantly different.

### 3.2 Effect of Bt maize, non-Bt maize, resistant check and commercial checks on maize damage and grain yield

There were significant differences between the four sets of hybrids (Bt hybrids, isolines, resistant check and commercial checks) in leaf damage, number of exit/entry holes, number of internodes tunneled, tunnel length and grain yield (Table 7). Bt maize had the lowest leaf damage, number of exit/entry holes, internodes tunneled and tunnel length followed by the resistant check, when infested with *C. partellus* and *B. fusca*. There were no significant differences between isolines and commercial checks in leaf damage and number of exit holes in all the three trials. However, the number of internodes tunneled were significantly lower in the isolines than in the commercial check in the second trial infested with *C. partellus* and the one infested with *B. fusca*. The length of tunnels was significantly lower in isolines than in commercial checks only in the first season of infestation with *C. partellus*. No significant differences were observed between the two in those other plantings. Bt hybrids had the highest yield followed by the resistant check in both seasons under *C. partellus* infestation; however, there were no differences in grain yield between Bt hybrids and their isolines under infestation with *B. fusca*.

**Table 7:** Pooled means of stem borer damage parameters and grain yield (±SEM) for the three sets of entries (Bt hybrids, isolines, conventional resistant, and commercial checks averaged across CFT and hybrids.

		Means (±SEM) of stem borer damage parameters and yield			
Entry	Leaf damage	Number of exit holes per plant	Number of internodes tunneled	Length of tunnel (cm)	Grain yield (kg/ha)
*Chilo partellus* (Season 1)					
Bt	1.1 ± 0.04a	0.2 ± 0.06a	0.2 ± 0.05a	0.5 ± 0.16a	7.9 ± 0.28d
Resistant check	3.3 ± 0.26b	2.9 ± 0.60b	1.7 ± 0.44b	3.5 ± 0.81b	6.0 ± 0.53c
Isolines	5.0 ± 0.17c	5.4 ± 0.65b	2.7 ± 0.26b	3.3 ± 0.31b	4.4 ± 0.33b
Commercial checks	4.9 ± 0.26c	5.5 ± 0.97b	2.6 ± 0.29b	4.2 ± 0.43c	3.1 ± 0.46a
F_3,65_	200.7	45.4	52.3	50.5	40.9
P-Value	<0.001	<0.001	<0.001	<0.001	<0.001
*Chilo partellus* (Season 2)					
Bt	1.1 ± 0.07a	0.1 ± 0.11a	0.1 ± 0.03a	0.1 ± 0.08a	10.6 ± 0.29b
Resistant check	4.3 ± 0.05b	3.9 ± 0.61b	1.5 ± 0.23b	4.0 ± 0.84b	7.6 ± 0.33a
Near Isolines	5.2 ± 0.11c	6.4 ± 0.55c	2.3 ± 0.17b	3.9 ± 0.33b	6.2 ± 0.44a
Commercial checks	5.5 ± 0.30c	6.3 ± 1.11c	2.6 ± 0.43c	3.7 ± 0.38b	6.2 ± 0.50a
F_3,65_	289.5	83.6	96.9	115.2	29.1
P-Value	<0.001	<0.001	<0.001	<0.001	<0.001
*Busseola fusca* (Season 1)					
Bt hybrids	1.9 ± 0.03a	0.1 ± 0.03a	0.04 ± 0.02a	2.1 ± 0.76a	8.9 ± 0.20b
Resistant check	2.4 ± 0.10b	1.4 ± 0.45b	0.9 ± 0.25b	11.2 ± 3.75b	7.6 ± 0.31a
Near Isolines	3.3 ± 0.09c	1.7 ± 0.24b	0.7 ± 0.14b	10.2 ± 0.76b	8.4 ± 0.21b
Commercial checks	3.1 ± 0.08c	1.5 ± 0.35b	1.1 ± 0.17c	9.5 ± 1.26b	7.7 ± 0.35a
F_3,65_	99.3	23.0	17.5	26.6	6.4
P-Value	<0.001	<0.001	<0.001	<0.001	<0.001

Means within a column for each trial followed by different letters are significantly different.

### 3.3 Recovery of larvae and pupae of *B. fusca* and *C. partellus* in Bt and non-Bt maize

The number of different stages of both species that were recovered was not analyzed to test differences between treatments as the numbers were very low (Table 8). *Chilo partellus* larvae were recovered in both Bt maize (hybrid 9) and two isolines (hybrids 2 and 10) in the first season planting, and only isoline 10 in the second season. *Chilo partellus* pupae were recovered in Bt maize (hybrid 9) in the first season and isoline 10 in the second season. *Busseola fusca* larvae were recovered only in the stems of isolines 4, 8, 12 and 14.

**Table 8:** Mean number (±SEM) of larvae and pupae of *Chilopartellus* and *Busseolafusca* recovered from Bt hybrids and their isolines following artificial infestation with *C. partellus* at Namulonge, Wakiso, and with *B. fusca* at Mubuku, Kasese, Uganda from 2014 to 2016 crop season.

	Mean number of larvae and pupae		
	*C. partellus*		*B. fusca*
**Entry code**	**Season 1**	**Season 2**	**Season 1**
Hybrid 1– MON810	0 ± 0	0 ± 0	0 ± 0
Hybrid 2– Isoline	0.05 ± 0.003	0 ± 0	0 ± 0
Hybrid 3 – MON810	0 ± 0	0 ± 0	0 ± 0
Hybrid 4 – Isoline	0 ± 0	0 ± 0	0.05 ± 0.03
Hybrid 5 – MON810	0 ± 0		0 ± 0
Hybrid 6– Isoline	0 ± 0		0 ± 0
Hybrid 7 – MON810	0 ± 0	0 ± 0	0 ± 0
Hybrid 8 – Isoline	0 ± 0	0 ± 0	0.05 ± 0.05
Hybrid 9 – MON810	0.08 (0.03)± 0.08 (0.03)	0 ± 0	0 ± 0
Hybrid 10 – Isoline	0.04 ± 0.04	0 (0.03) ± 0 (0.03)	0 ± 0
Hybrid 11– MON810	0 ± 0	0 ± 0	0 ± 0
Hybrid 12– Isoline	0 ± 0	0 ± 0	0.05 ± 0.03
Hybrid 13– MON810	0 ± 0	0 ± 0	0 ± 0
Hybrid 14– Isoline	0 ± 0	0 ± 0	0.10 ± 0.07

Figures in parentheses are number of pupae recovered from the different entries.

## 4 Discussion

In this study, we demonstrated the efficacy of MON810 maize in controlling *C. partellus* and *B. fusca* on maize in Uganda. The Bt maize (that was artificially infested with *C. partellus* and *B. fusca* showed significantly lower leaf damage, fewer exit holes, and reduced tunneling by stem borers when compared with the corresponding isolines, conventional resistant check, and the commercial non-transgenic reference maize varieties. As a result, the Bt maize produced significantly higher grain yield (29.4–80.5%) than the isolines and the commercial non-transgenic maize varieties. *Chilo partellus* larvae and pupae were recovered in isoline, resistant check, and commercial varieties, but in only one of the Bt hybrids, while *B. fusca* larvae and pupae were only recovered in the resistant check, isolines, and commercial varieties. Reduced stem borer damage (leaf damage, number of exit holes, and tunnel lengths) in the Bt maize as compared to the non-transgenics showed and confirmed that transgenic Bt maize (MON810) is effective against *C. partellus* and *B. fusca*. Similar results were reported in Kenya for *B. fusca* in the laboratory and *C. partellus* in CFT trials (Tefera et al., 2016; Tende et al., 2010).

The high expression of Bt protein in maize leaves as reported by Nguyen and Jehle (2007) explains the success of Bt maize in managing maize stem borers. The use of different promoters in commercial Bt maize hybrids leads to differential expression of toxins in different plant tissues (Dutton et al., 2003; Van Wyk et al., 2009). The maize events MON810 and Bt11 contain the cauliflower mosaic virus (CaMV) 35 S promoter, which results in toxin expression in the leaves, stem, roots, and kernels at all maize growth stages (EPA, 2001). If differences occur in Bt-toxin concentrations within a plant, control success may be compromised as is the case when larvae feed on silks and kernels with a lower toxin concentration, and later enter the stems as 3rd instars. This may lead to development up to the adult stage. Indeed, van Rensburg (2001) reported successful control of *B. fusca* during the vegetative stages because of high protein expression, and survival of *B. fusca* 1st instar larvae when fed on maize silks with lower toxin levels.

Grain yields realized from MON810 Bt hybrids were higher than those of the isolines, resistant check and commercial varieties. This shows that protection from stemborer damage resulted into higher grain yield. In addition to guarding against grain yield losses, MON810 Bt maize can also limit field infections by *Aspergillus* spp., thereby reducing aflatoxin contamination and contributing to food safety and reducing risks caused by aflatoxins (Schulthess et al., 2002; Sétamou et al., 1998). A similar study by Kocourek and Stará (2018) on the European corn borer in the Czech Republic reported reduced damage and incidence of *Fusarium* species on Bt maize (MON810), with a corresponding yield advantage of 15% over the non-Bt maize. Bt crops can be a useful component of integrated pest management (IPM) systems to protect the crop from targeted pests (Mabubu et al., 2016).

We observed some cases of exit holes and stem tunneling in Bt maize hybrids infested with either species of stem borers, and one pupa in Bt maize infested with *C. partellus*, implying incomplete control of *C. partellus* by Bt maize. This may be because of a window of opportunity for successful feeding and survival of later generation neonates and second instar larvae on silks, which were postulated to have a reduced concentration of the Bt toxin (van Rensburg, 2001). We also observed higher leaf damage and tunneling by *B. fusca* in Bt hybrids suggesting that *B. fusca* requires higher amount of Bt plant tissue to cause mortality. The more extensive tunneling also implies that the pest can easily develop to adulthood once it gains entry into the stem, especially that Bt maize plants were reported to have lower concentration of the toxins in the stalk (Nguyen and Jehle, 2007). The lack of complete control is not consistent with previous studies that showed 100% mortality of *C. partellus* (Singh et al., 2005; van Rensburg, 1998). This may be explained by observations of other authors who reported that the expression of the protein varies with plant parts, variety, environmental conditions, season, and phenology of the plant (Nguyen and Jehle, 2007; Székács et al., 2010; Trtikova et al., 2015).

Adopting Bt maize could help farmers in Uganda to guard against grain yield losses associated with stem borers, thereby improving household food security, incomes, and livelihoods. Growing Bt maize would also eliminate/reduce the costs associated with the use of insecticides, and lessen the dangers to humans and the environment as a result of pesticide misuse/overuse. Sustainable use of Bt maize will, however, depend on the development of products that offer season-long protection against different strains of the pests, regulatory compliance, and product stewardship, among other requirements. Product stewardship means that everyone involved in the product life cycle – innovators, scientists, and technology users – is accountable for ensuring that the products are safe and socially and environmentally responsible (Mbabazi et al., 2020).

The incursion by fall armyworm (FAW) (*Spodoptera frugiperda*) (JE Smith) in Africa (Goergen et al., 2016; Otim et al., 2018) is a new challenge on the continent. Originally, known to be restricted to the Americas, the FAW has become the most damaging pest of maize in Uganda, and many sub-Saharan Africa countries (Day et al., 2017; FAO, 2018). Though event MON810 offers partial control for FAW (Prasanna et al., 2018), it was not developed to control this pest. The use of maize with MON810 should be considered primarily as a tool to manage stem borers, with additional Bt events needed for more effective control of both stem borers and FAW. This should be accompanied by a comprehensive insecticide resistance management strategy that forms an integral part of Bt deployment. The evolution of dominant resistance to Cry1Ab protein in *B. fusca* in South Africa (Campagne et al., 2013) calls for robust resistance management strategies, including the use of integrated pest management (Campagne et al., 2013) and the use of other Bt toxins in maize with different receptors in the target insects.

## 5 Conclusion

Our study has demonstrated that Bt maize (MON810) with Cry1Ab was effective in controlling *C.* *partellus* and *B. fusca* in our trials in Uganda in 2014–2016. Bt maize protected against leaf damage and limited stem borer entry into maize stems, resulting in 29.4–80.5% higher yield than in the non-transgenic hybrids. Bt maize has potential to help Ugandan maize farmers produce high-quality grain with greater yield and less reliance on insecticides, and thus enhancing food security. This study was conducted in only two locations because of regulatory requirements. Additional studies may be needed in multiple locations to capture representation from different populations of the stem borers. Such studies could be part of the testing in national performance trials for variety registration and commercialization.

## Author contribution

**Michael H. Otim**: Conceptualization, methodology, formal analysis, investigation, writing original draft, writing – review and editing – visualization, supervision, project administration; **Grace Abalo**: Investigation; methodology, writing – review and editing; **Godfrey Asea**: Conceptualization, investigation, writing-review and editing, supervision, project administration, fund acquisition; **Julius Pyton Sserumaga**: Investigation, formal analysis, writing – review and editing; **Simon Alibu**: Investigation, writing – review and editing; **Stella Adumo**: Investigation, data curation, supervision, writing – review and editing; **Jane Alupo**: Investigation, data curation, supervision, writing – review and editing. **Stephen Ochen**: Investigation, data curation, supervision, writing – review and editing; **Tadele Tefera**: Conceptualization, investigation, writing – review and editing; **Anani Yaovi Bruce**: Investigation, writing original draft, writing – review and editing; **Yoseph Beyene:** Investigation, writing – review and editing; **Evans Njeru**: Writing – review and editing, supervision; **Barbara Meisel**: Methodology, resources, writing – review and editing; **Regina Tende**: Methodology, investigation, writing – review and editing; **Francis Nang'ayo**: Writing – review and editing, supervision; **Yona Baguma**: Writing – review and editing, supervision, project management; **Stephen Mugo**: Conceptualization, methodology, resources, investigation, writing – review and editing, supervision, project administration, fund acquisition; **Sylvester O. Oikeh**: Conceptualization, methodology, resources, investigation, writing – review and editing, supervision, project administration, fund acquisition.

## Declaration of competing interest

The authors declare that they have no known competing financial interests or personal relationships that could have appeared to influence the work reported in this paper.
